# Experiences with Negative Behavior and Incivility: Perspectives of Unlicensed Assistive Personnel and Registered Nurses

**DOI:** 10.3390/nursrep14030127

**Published:** 2024-07-16

**Authors:** Diana Layne, Christina Beall, William T. Bryant, Lynnette Morris, Heather Craven

**Affiliations:** 1 College of Nursing, Medical University of South Carolina, Charleston, SC 29425, USA; 2 Department of Nursing, University of South Carolina-Beaufort, Bluffton, SC 29909, USA; cabeall@uscb.edu; 3 Ralph H. Johnson VA Health Care System, Health Equity and Rural Outreach Innovation Center, Charleston, SC 29425, USA; william.bryant2@va.gov; 4 Department of Psychiatry and Behavioral Sciences, Medical University of South Carolina, Charleston, SC 29425, USA; 5 MUSC Health, Medical University of South Carolina, Charleston, SC 29425, USA; morrislm@musc.edu (L.M.); cravenh@musc.edu (H.C.)

**Keywords:** negative behaviors, incivility, lateral violence, bullying, nursing

## Abstract

Healthcare professionals experience negative behaviors such as incivility from various sources within the hospital environment. However, little is known regarding the experience of unlicensed assistive personnel with these behaviors. Using a cross-sectional survey design, the research team aimed to examine the presence, sources, and impact of negative behaviors among registered nurses and unlicensed assistive personnel within a US hospital. Descriptive and inferential statistics were used to analyze quantitative data, while thematic analysis was used to analyze the qualitative responses. A total of 309 participants completed the survey, and 135 participants responded to three qualitative questions. Most respondents identified inadequate staffing/resources to handle workload (87%) and job stress leading to loss of control over behavior as contributing factors to lateral/vertical aggression in the work environment (71%). Impacts of negative behavior on job performance were related to both personal well-being and the work environment. Demoralization was identified as a common consequence of negative behaviors for individuals and within the work environment. The results suggested that registered nurses, unlicensed assistive personnel, and nursing leadership may benefit from system-wide approaches addressing negative behaviors such as incivility within the clinical environment. Specifically, efforts and policies aimed at aiding clinicians in responding to negative behaviors could potentially improve the clinical environment.

## 1. Introduction

Acute care hospitals continue struggling to recruit and retain adequate staff to support the provision of safe, equitable, clinical care for hospitalized individuals. One critical relationship that exists within acute care hospital units is that of the registered nurse (RN) and unlicensed assistive personnel (UAP). Examples of UAPs include certified nursing assistants (CNAs), patient care technicians (PCTs), nurse aides, nurse techs, and nurse auxiliaries. This dyad is responsible for providing most of the direct patient care with a portion of direct care provided by other allied health professionals (e.g., physical therapy, occupational therapy, respiratory therapy, etc.). Evidence suggests nurses experience various negative behaviors within the work environment such as incivility [1], bullying [2] and violence [3]. Primary sources of these behaviors include patients/families, peers, supervisors, and those with prescribing authority (e.g., physicians and nurse practitioners) [4]. However, the experiences of UAPs with negative behaviors remains understudied. 

Negative or disruptive behaviors are interactions between staff, coworkers, and/or patients that negatively impact patient care [5]. The umbrella of negative behaviors is intentionally broad, spanning from the more passive and ambiguous (e.g., incivility and conflict) to more overt and intentional (e.g., bullying and violence) [6,7]. Negative behaviors generally have been related to unfavorable patient outcomes, noxious work environments, deteriorated interpersonal relationships, poor self-esteem, reduced productivity, and intention to leave [8,9]. In healthcare settings, both nurses and unlicensed personnel are experiencing incivility from other healthcare coworkers, as well as patients for whom they are providing care [10]. However, incivility’s impact is not unique to healthcare, with a recent meta-analysis finding a significant effect of workplace incivility on intent to leave across broader industries [11]. 

One review of nursing studies found bullying was consistently related to increased stress and burnout, as well as reduced productivity and absenteeism [12]. Physical health consequences of experiencing bullying include gastrointestinal symptoms, hypertension, headaches, eating disorders, and sleep disturbances [13]. As with stress and burnout, bullying exposure increases the risk of staff turnover [14]. 

### Purpose and Research Questions

The purpose of this research was to examine the presence and sources of negative behaviors among nurses and unlicensed assistive personnel working at an academic health science center using the Negative Behaviors in Healthcare Survey (NBHC) [7] and Nursing Incivility Scale (NIS) [4]. Additionally, perspectives related to positively resolved uncivil experiences, impacts of negative behavior on job performance, and strategies and tools to promote a culture of civility within the work environment were also solicited using open-ended questions.

## 2. Materials and Methods

A cross-sectional descriptive survey was conducted from late September through October 2021. The online survey utilized the online survey platform REDCap for data collection and management hosted at Medical University of South Carolina [15,16]. The survey included demographic questions, the NBHC, the NIS, and the three open-ended questions. Participants reported data anonymously and had the ability to skip instrument items and open-ended survey questions if desired. Participants did not receive compensation for their time to complete the survey.

### 2.1. Sample and Recruitment 

Due to the descriptive nature of this study a convenience sample of registered nurses (RNs) and UAPs were eligible to participate if they had been employed for a minimum of 30 days and had the ability to complete an online survey in the English language. The study was approved by the local Institutional Review Board (PRO00110431). Potential participants were recruited via posted flyers within the clinical areas and an email invitation from the chief nursing officer with subsequent email reminders to participate in the study.

### 2.2. Measures

Upon review of the study information and confirmation of eligibility, participants anonymously answered demographic questions: age, sex, role, shift, race, ethnicity, education, certification status, the NBHC, and the NIS. Respondents were then asked three open-ended questions related to positively resolved uncivil experiences, impacts of negative behavior on job performance and strategies, and tools to promote a culture of civility within the work environment. 

#### 2.2.1. Negative Behaviors in Healthcare Survey

The NBHC is a 25-item self-report survey using Likert responses across five subscales including contributing factors to negative behavior, fear of retaliation, frequency and use of lateral/vertical aggression, and perceptions of the seriousness of lateral/vertical aggression. Lateral aggression was defined as “occurring between colleagues at the same power level within healthcare (e.g., staff RN to staff RN; resident physician to resident physician)”, while vertical aggression was defined as “occurring between colleagues at different power levels within healthcare. It may be directed downwards (abuse of legitimate authority, e.g., manager to subordinate, attending physician to resident physician) or upwards (abuse of informal power, e.g., subordinate to manager)” [7]. Finally, disruptive or inappropriate behavior was defined as “behaviors such as eye rolling and other nonverbal messages, rude remarks, name calling, condescending communication, infighting, deliberately not helping team members, not passing along important information, deliberately setting someone up to fail/get in trouble/look bad, talking behind a coworker’s back, spreading rumors, scapegoating, breaking a confidence, excluding, silent treatment, not responding to questions/comments/pages, hanging up phone abruptly before problem is resolved, criticizing excessively, cyber abuse, making unfair assignments, withholding opportunities” [7]. Internal consistency (Cronbach’s alpha) for subscales in prior studies ranges from 0.62 to 0.92 [7]. 

#### 2.2.2. The Nursing Incivility Scale

The NIS is a 43-item instrument using Likert responses across five sources of incivility (general, nurse, supervisor, physician, and patient/family) across eight behavior categories including hostile climate, inappropriate jokes, inconsiderate behavior, gossip and rumors, freeriding, abusive supervision, lack of respect, and displaced frustration. Incivility is defined as a “low intensity behavior with ambiguous intent to harm that violates workplace norms of mutual respect” [4]. With permission from the author the NIS was modified to include an additional 10 items specific to UAPs based on the initial items measuring incivility from nurses. The NIS instrument is available separately [4]; the ten items referring to interactions with other nurses were duplicated and the instructions revised for participants to “describe your interactions with other unlicensed assistive personnel (patient care technicians, therapy assistants, medical assistants, etc.)” when responding to those items. The original items referring to experiences with other nurses were also retained. Internal consistency for the NIS subscales in prior studies ranges from 0.81 to 0.94 [4].

#### 2.2.3. Levels of Leadership

For the purposes of this research, leaders and supervisors were considered those with accountability for staff and patients on clinical units. Administrators were those responsible for the management of nurse leaders and supervisors and this included other members of hospital executive leadership (e.g., chief nursing officer, hospital president, etc.).

### 2.3. Data Analysis

Descriptive statistics were calculated for quantitative data using the Statistical Package for the Social Sciences (SPSS) v. 28. The data were reviewed for missingness, and the results were analyzed using pairwise deletion to maximize the use of available data. Mann–Whitney U tests were used to compare NBHC and NIS scale scores between RNs and UAPs due to the nonnormal distribution of the subscale scores between groups. The frequency distributions of individual item responses were also reviewed. Thematic analysis [17] was utilized to analyze participant responses for the three open-ended questions. Two research team members independently analyzed responses assigning preliminary codes. The consensus of the entire research team was used to resolve any disagreement between initial coders. The preliminary codes were then reviewed and condensed into final codes. Final codes were then organized into themes by the research team. Responses that did not address the questions asked were excluded from the analysis. Peer debriefing among the research team was used to ensure trustworthiness and maintain rigor of qualitative data. Due to the anonymous nature of the survey results, member checking with participants to ensure the accuracy and validity of interpretations of qualitative data was not possible. The qualitative responses were also analyzed to determine the involvement of new nurses or staff members, source of incivility (e.g., patient, family, peer, etc.), event type, and resolution for experiences of incivility that were positively resolved. Responses related to the impacts of negative behavior on the work environment were analyzed to identify contributing factors, impacts on personal well-being, and impacts on the work environment. Finally, the qualitative responses were analyzed to identify strategies and tools to promote civility within the work environment.

## 3. Results

A total of 477 participants opened the survey with 309 participants (245 RNs, 64 UAPs) completing the survey and 136 participants (110 registered nurses, 25 unlicensed assistive personnel, and 1 unreported role) responding to the open-ended questions. The overall response rate based on staff employed in clinical units as of October 2021 was around 11.4% (309/2711). Most participants were white females with a mean age of 39.8 (SD 11.08). Most RN participants reported having a bachelor of science degree compared with a high school diploma for UAP participants. Participants reported a mean 13.6 (SD 10.1) years working in healthcare and a mean 9.3 (SD 8.7) years in their professional role. Table 1 describes participant demographic characteristics by professional role (RN or UAP).

### 3.1. Negative Behaviors in Healthcare (NBHC)

Statistically significant differences between RN and UAP participants were observed across three of the five NBHC subscales. These included contributing factors (U = 5690.5, *p* = 0.02), observing aggression (U = 5280.5, *p* = 0.001), and recipient of aggression (U = 5982.5, *p* = 0.02). The Mann–Whitney U results by role are presented within Table 2. Analysis of frequency distributions by individual question revealed 87% of respondents identified inadequate staffing/resources to handle workload, and 71% identified job stress leading to loss of control over behavior as contributing to lateral/vertical aggression in the work environment. Further, many participants (51%) reported peers were not willing to intervene compared with 41% of leaders not willing to intervene as additional contributing factors. Almost half of respondents reported observing lateral aggression (48%) at least monthly compared with 27% of respondents who reported being the recipient of lateral aggression at least monthly. In contrast, vertical aggression downward from leadership to healthcare professionals was observed by 38% of the participants. In terms of safety of retaliation, 22% reported feeling safe to report lateral aggression, while 27% reported feeling safe to report vertical aggression. When reviewing questions related to the seriousness of lateral/vertical aggression, greater than 50% of participants agreed this was a serious or very serious issue. Finally, 23% of participants reported leaving a position due to vertical aggression compared with 22% of participants who reported leaving a position due to lateral aggression. Internal consistency of the NBHC subscales was calculated using Cronbach’s alpha and ranged from 0.80 to 0.95, which is consistent with acceptable values of 0.80 or greater [18].

### 3.2. Nursing Incivility Scale (NIS)

Statistically significant differences between RN and UAP participants were observed across two NIS behavior subscales. These included gossip and rumors (U = 3934.5, *p* = 0.01) and abusive supervision (U = 3901.5, *p* = 0.01). Additionally, statistically significant differences were also identified in experiences with incivility from UAPs (U = 3643.5, *p* = 0.03) and physicians (U = 3372.2, *p* ≤ 0.001). Interestingly, frequency distributions of individual items revealed 66% of participants reported nurses “gossip about one another” compared with 53% of participants reporting UAPs “gossip about one another”. Moreover, over a third of participants reported physicians “are condescending to me” and being treated as “my time is unimportant”. Finally, three fourths of participants reported patients and families “have taken their frustrations out on nurses” and “shown they are irritated or impatient”. While statistically significant differences were not reported between RNs and UAPs in general, incivility individual item analyses revealed 88% of participants reported “people make jokes about minority groups”, while 71% reported “people make jokes about religious groups”. The internal consistency of the NIS subscales was calculated using Cronbach’s alpha and ranged from 0.75 to 0.94.

## 4. Qualitative Results

The analysis of the responses from the first question regarding examples of positively resolved uncivil experiences revealed four themes including active communication, passive strategies, leadership engagement, and unresolved. While analyzing the responses related to negative behaviors’ impacts on job performance, the responses were clearly delineated between impacts on personal well-being and impacts on the work environment. Additionally, many responses included information related to contributing factors impacting participants’ ability to perform job responsibilities. Examples of these contributing factors included an uncivil environment, degraded teamwork, and aggression, and those responses without clear contributing factors were classified as unknown. Five themes emerged related to the impacts of negative behaviors on personal well-being including demoralization; physical, psychological, and emotional symptoms; distraction and disengagement; interpersonal interference; and no impact, while five themes emerged as impacts of negative behavior on the work environment including interpersonal interference, demoralization, disengagement, negative work environment, and no impact. Finally, four themes emerged as strategies to promote a culture of civility within the workplace including a psychologically safe environment; system-level strategies; leadership behaviors; and diversity, equity, inclusion, and belonging. The subsequent sections describe each of these themes in further detail. 

### 4.1. Sources of Incivility and Involvement of New Nurses

Most participant responses described uncivil encounters among and between RNs and UAPs. Patients, families, and physicians were also identified by RNs and UAPs as sources of incivility. Responses describing incivility with healthcare providers outside of the RN/UAP category were reported less frequently in the qualitative responses. In this portion of the survey, supervisors were the least commonly recognized source of incivility for RNs and UAPs. Some participants indicated that a supervisor was directly involved in the effective management and resolution of an uncivil encounter. It is interesting to note that new RNs and UAPs were not recognized in the qualitative data as having an increased level of uncivil events. 

### 4.2. Positively Resolved Uncivil Experiences

Seventy-eight participants responded to the item “Describe one time when you experienced incivility and it was resolved in a positive way”. Most described incidents of incivility (n = 28) or bullying (n = 26). Other types of incidents included conflict (n = 4) and discrimination (n = 2). Only six of the responses described incidents involving new nursing staff. Seventeen participant responses lacked the detail required to classify the type of incivility experienced. The most common sources of incivility identified were RNs or UAPs (n = 24) or patients and family members (n = 21). The responses described one of four types of resolution: active communication (n = 36), passive strategies (n = 16), leadership engagement (n = 16), or remained unresolved (n = 10). 

#### 4.2.1. Active Communication

Descriptions of the use of active communication to resolve incivility involved recognition of the situation and advocating for themselves or others using direct communication. For example, one participant described the following situation.

*A nurse spoke down to me and questioned the integrity of my work with a patient. This problem was resolved between the nurse and I after we took a moment. We both were able to admit our wrongs and find the cause of our miscommunication. I sincerely feel like it brought us closer as a team.*

*(UAP 237)*

At times, active communication involved other interprofessional team members setting expectations of appropriate communication. For example,

*I had a patient calling me the “N word”. The primary team came to the bedside and had a conversation with the elderly patient about her inappropriate language.* In the English language, the “N word” refers to a highly offensive racial slur to dehumanize or devalue individuals of African American descent.

*(RN 75)*

In response to active communication about the incident, the source often acknowledged the inappropriate behavior and apologized to the recipient.

#### 4.2.2. Passive Strategies

Passive strategies to resolve incivility described by participants included responses such as “I just walk away” (RN 259) or communicating indirectly by sharing the incident with peers or staff leaders.

*Literally every shift I encounter either a rude RN, rude PCT [Patient Care Technician] and (most likely) a rude physician. I ignore it. Not sure if that’s resolution.*

*(RN 206)*

#### 4.2.3. Leadership Engagement

Leadership engagement in the resolution of incidents was either direct or indirect. Some participants described situations where leadership engagement in the situation led to resolution. For example, an RN in a staff leader role described the following incident.

*A patient showed hatred and used offensive language toward a colleague of mine who is gay. The colleague left upset and embarrassed by the patient’s comments. I calmly but firmly called the patient out for his treatment of my colleague. He acknowledged he was in the wrong and apologized and also apologized to the colleague he had hurt.*

*(RN 219)*

On the other hand, leadership engagement may be more indirect or used as a teaching moment for the staff. In one incident, the participant described the leader’s indirect response as a positive resolution.

*I was spoken to rudely by a coworker. My manager talked about lateral violence and bullying to the group as a whole. I felt this helped because it was not singled out to one person, and everyone knew how important it was.*

*(RN 233)*

#### 4.2.4. Unresolved

Unfortunately, not all participants found resolution for incivility. One participant described an incident as “*never was resolved in a civil way. It was like management didn’t care*”. (*RN 122*) In some cases, the lack of resolution led to participants leaving the workplace where they experienced the incivility. One participant explained the following situation.

*I worked on a [medical surgical unit] here and left just about 10 months ago when the manager and her “friends” constantly belittled me, treated me like I was less than dog poo on their shoes. So, I left, and it was the best decision I have ever made. Almost left [the organization] all together because of her; I have 22 years of service vested here. I was treated badly for over 2 years.*

*(RN 95)*

While some participants reported unresolved incivility, most positive resolutions reported were facilitated when the RNs and UAPs used direct communication techniques with one another. Descriptions of direct communication approaches between the involved parties suggest a willingness to address issues as they arise directly with those involved. 

## 5. Negative Behavior Impacts

One hundred and sixteen participants responded to the question “*How has your ability to perform your job been impacted by negative behavior (lateral/vertical aggression, incivility, bullying, etc.) in the work environment?*” Each response was analyzed to identify factors that contributed to the impact on job performance, the impact on the participant’s well-being, and the impact the incident had on the work environment. Participants described an uncivil environment as “*negative behavior and blaming … lots of gossip, just makes work one of those places you want to get in and get out and not get involved*” *(RN 51)* and “*I have dealt with bullying by co-worker that deeply impacted my morale and job performance*”. (RN 276) 

### 5.1. Contributing Factors

The most common contributing factors described were an uncivil environment (n = 58), degraded teamwork (n = 25), and aggression (n = 7). For some participants the uncivil environment had significant impacts on their careers. For example, one participant described the impact as follows.

*I couldn’t wait to call out from work. I hated working there and being treated so badly. I cried almost every day on the way to work and the pandemic made it even worse. There were times I became very suicidal, thinking of wrecking my car on the way to work. I‘m terrified of pain, so I never did it and Thank God every day for my new home and unit at the [new department]. I LOVE it here!!!!!*

*(RN 95)*

When degraded teamwork contributed to performance issues, participants described situations such as “*I‘ve seen colleagues not report or question something because they fear the reaction of the provider*”. *(RN 262)* Ultimately, degraded teamwork threatens patient safety as described by the following participant’s statement.

*Creates stress and takes more effort to remain focused on the job at hand. Creates a very unpleasant work environment. Creates great inefficiency in getting the job done, communicating important information, keeping the patient safe. Creates an environment of intimidation.*

*(RN 167)*

When describing incidents involving aggression, participants described it as “*The aggression I have witnessed are contributing factors for re-thinking my placement within this organization*”. *(RN 307)* The impact of the aggression was described by one participant as follows.

*Whenever this happened (not currently going on), I was depressed to the point of tears, calling out sick [due to] panic attacks, and actually dreading coming into work so much that I felt nauseated. I became hypervigilant and nervous when the person in question came near me. I was basically non-functional in my job role due to the vertical violence.*

*(RN 477)*

Unlicensed assistive personnel reported similar experiences of aggression. One UAP described the impact of aggression as follows.

*As a victim of vertical aggression, my ability to perform my job was greatly impacted. I experienced severe anxiety when I saw myself scheduled with that specific nurse. I lost all confiden[ce] in my abilities and dreaded coming to work.*

*(UAP 185)*

Although it was not always possible to identify contributing factors, participants were still able to describe the impacts of the behavior, such as “*Made me feel worthless and incapable of myself and job*” *(RN 419)* and “*It made me want to quit nursing*” *(RN 252)*.

### 5.2. Impact on Personal Well-Being

In terms of the impact of the negative behavior on the participants’ well-being, common identified themes included demoralization (n = 31), psychological and emotional symptoms (n = 24), distraction and disengagement (n = 16), and interpersonal interference (n = 12). Demoralization as used here involves the loss of confidence, enthusiasm, and hope. Participants described the impact of demoralization on their well-being as “*Lack of support from someone that is supposed to be a leader but who shows favoritism makes it hard to work with them*”. *(RN 7)* One participant described a profound impact of demoralization on their well-being, both in and out of the workplace,

*Prior manager targeted and bullied me daily. It lowered my self-esteem. It changed my entire personality. It stressed my job performance as well as home/family life.*

*(RN 429)*

Participants also described psychological and emotional symptoms they experienced, such as “*it is very distracting, irritating, and unpleasant*”. *(RN 87)* For others, symptoms were described as follows:

*I no longer enjoy coming to work and my anxiety has increased substantially. I am unable to perform as well as I used to because I no longer have the help and support from my coworkers.*

*(UAP 186)*

Incivility also led participants to experience distraction and disengagement. For example, one participant described being “*less likely to be quick to communicate concerns to those that don’t value my assessments and opinions of patient status. Second guessing myself*” *(RN 182)*.

Another described the impact as “*I‘m more guarded and less fulfilled in my current position lately*”. *(RN 100)* When the incivility interfered in interpersonal relationships, participants described it as “*Communication begins to breakdown as well as the trust*” *(RN 389)*.

### 5.3. Impact on Work Environment

Similar themes emerged when participants described the impacts of incivility on the work environment, including interpersonal interference (n = 35), demoralization (n = 31), negative work environment (n = 15), and disengagement (n = 11). Interpersonal interference resulted in a work environment where participants reported being “*Less likely to voice concerns or ask for help*”. *(RN 27)* The ultimate impact on the work environment was described as follows.

*Negative/apathetic behavior has left us short staffed—unsafe staffing. Negative behavior has divided staff and left many people fending for themselves causing unsafe situations.*

*(RN 113)*

Demoralization impacted the work environment, leading a participant to “*question everything, I do. I‘m having a hard time making a decision in fear of making a decision that will upset others and not making a decision based on what’s best*” *(RN 202)*.

Another participant described the impact as “*I feel like I don’t fit in, inadequate and have considered quitting. It is intimidating to be bossed around and spoken to like I am sub-standard considering my years of experience*” *(RN 168)*.

Incivility was attributed to creating a negative work environment as follows:

*Nurses are afraid to page providers about concerns because of being looked at as stupid, treated as if they are a bother, etc. and this puts patient safety at risk because then nurses are less likely to reach out to a provider with an issue for fear of their reaction.*

*(RN 55)*

*I’ve seen colleagues not report or question something because they fear the reaction of the provider.*

*(RN 262)*

For other participants, incivility led to disengagement, which “*takes focus off the patient and safety. It is a huge waste of time*”. *(RN 87)* One participant’s response describes the impact as follows:

*Dropped my hours due to scheduler and select staff bullying and favoritism. I am one of the more senior nurses on the unit but decided my mental health was more important.*

*(RN 290)*

RNs and UAPs reported that a work environment riddled with incivility had a negative impact on their job performance. Incivility reportedly caused varying levels of distraction, demoralization, and disengagement that had a direct and negative impact on job performance. Additionally, RNs/UAPs reported that they suffered from psychological and mental health issues related to a negative work environment impacting their ability to provide safe care. Another theme that emerged was a decrease in communication and subsequent decreased engagement among team members. When team members are disengaged and develop a lack of confidence, positive communication patterns can suffer. 

## 6. Promoting a Civil Culture

The final open-ended question asked participants, “What strategies and/or tools do you need to promote a culture of civility within the workplace?” The themes identified in the 113 participant responses included psychological safety (n = 35); system-level strategies (n = 33); leadership behaviors (n = 23); and diversity, equity, and inclusion behaviors (n = 18).

Psychological safety responses focused on creating an environment where participants felt safe to respond appropriately to incivility or other events. For example,

*The security and knowledge that I can report such occurrences without reprimand.*

*(RN 477)*

*Using effective communication, positive phrases and comments when talking with each other. Mistakes are not to be seen as punitive or negative, rather areas for improvement for better outcomes.*

*(RN 200)*

*Treat others with respect—you don’t have to like someone to be kind. We are here to help our patients, and that should always come first.*

*(RN 250)*

Participant suggestions for system-level strategies to respond to incivility focused on implementing no-tolerance policies and dealing with systemic issues that promote incivility. One participant suggested the following:

*A more positive manager. [For the Human Resources Department]/Upper Management to listen when concerns are brought to them about manager/team members. Leadership needs to have a no tolerance policy to incivility/bullying.*

*(RN 450)*

On the other hand, some participants identified situations that are contributing to a work environment where incivility occurs, such as the following:

*floating* nurses [transferring nurses to work in areas outside their designated clinical unit] *to areas they are not comfortable and taking staff away from adequately staffed units is creating and perpetuating the problem. It is a viscous cycle that over my 33 years of being a nurse continually repeats itself. We need to examine why we do not have enough nurses and why they leave the bedside nurse position. There needs to be more VALUE on the bedside nurse so that nurses are not looking for ways to leave these positions.*

*(RN 105)*

Leadership behaviors participants described to improve the work environment focused on employee interactions and response to patient/family behaviors:

*Good leadership from management, dealing directly with problematic employees that create an unprofessional culture.*

*(RN 437)*

*A strong management team that will advocate for us.*

*(RN 98)*

*I need administration to actually care and do something when patients and their families are being verbally abusive.*

*(UAP 161)*

With the current emphasis on improving diversity, equity, and inclusion in healthcare, suggestions also included how to leverage those behaviors to improve the work environment. One participant reported the following:

*When any employee comes into the unit, regardless of title, they should possess positive body language and greet others. There also needs to be a sense of cultural competence and discriminatory practices must be addressed. Our patients are watching, and we must set the standard.*

*(RN 66)*

Finally, the power differential between care team members requires attention. One example provided by a participant stated, “*I need doctors to respect nurses and [technicians], how they speak to them and treatment in general*” *(RN 252)*.

Overall, psychological safety within the work environment requires a shared belief held by interprofessional team members that it is acceptable to take risks, to express their ideas and concerns, to speak up with questions, and to admit mistakes without fear of negative consequences. The suggestions provided by the RN and UAP participants indicate a need to attend to and shift organizational culture by providing staff and leaders with opportunities to improve communication skills and address diversity, equity, and inclusion within the organization. Systemic healthcare issues perceived as contributing to an environment of incivility, such as staffing shortages, the value of bedside nurses, and tolerance of negative behaviors from leaders, as well as patients and families, must be reframed and requires innovative strategies to create a civil work environment free of violence.

## 7. Discussion

The study findings revealed that RNs and UAPs reported statistically significant differences in three of the five NBHC subscales and in two of the NIS subscales. RNs more often than UAPs reported observing factors that contributed to incivility in the workplace such as rude behavior, personality clashes, power/control issues, and a lack of leadership and/or peer intervention when incivility occurred. This was consistent with findings from Jabar and colleagues who examined the association between horizontal violence among Jordanian nurses and patient safety culture [19]. RNs also reported observing aggression and being the recipient of aggression at higher rates than UAPs. It is possible that RNs are more likely to recognize less overt uncivil behaviors when they occur and are better able to label them as such. It is also possible that RNs are more adept at identifying covert acts of aggression when they occur. Less obvious acts of aggression such as rolling of the eyes, cutting others off, and being dismissive are often subtle and may not be readily identified by UAPs as aggression. In contrast, the qualitative responses indicated that UAPs also experienced impacts from aggressive behavior.

In the NIS subscales, gossiping and rumor spreading occurred at higher rates between RNs when compared with gossiping/rumor spreading between UAPs. Additionally, RNs reported higher rates of uncivil encounters during interactions with UAPs and physicians than those reported by UAP participants. Once again, it is possible that RNs were more likely to recognize the subtle acts of incivility more readily than the UAP participants in this study. Another consideration is that job responsibilities may influence this as RNs typically interact with physicians more often than UAPs, which might explain the differences in reporting uncivil behavior. Despite the statistically significant difference in reporting, gossip was identified as a common problem for both RNs and UAPs with over half of participants reporting both RNs and UAPs gossip about peers; the qualitative response also supported the presence of gossip within the clinical environment. This suggests the potential for the presence of the adverse effects of gossip. Two potentially harmful effects of gossip within medicine include the propagation of harmful misinformation and the emphasis of malicious commentary [20]. Further, evidence suggests gossip is positively related to burnout and suboptimal care and negatively related to patient safety and job engagement [21]. The qualitative results were consistent with these findings.

Additional survey responses indicate that RNs and UAPs reported similarities in their experiences with incivility. The most common source of incivility reported by both RNs and UAPs was patients and families. This finding was consistent with a recent systematic review indicating patients as a common source of incivility [22]. Our results revealed 78% of participants were recipients of patient and family frustrations, which was consistent with a recent American Nurses Association Workplace survey citing patients, families, and peers as the most common sources of incivility within the clinical environment [23]. Numerous publications describe the ongoing presence and frequency of incivility faced by nurses and other healthcare workers [8,18,22,24,25]. Our findings were consistent with these studies and supported that overall incivility continues to be a significant issue impacting the healthcare work environment, communication patterns among providers, patient care, and the overall mental and physical health of nurses and UAPs. Additionally, it is well established that an uncivil and hostile work environment can have a profoundly negative impact on nurses’ work satisfaction, intent to leave, and patient outcomes [8,24,25,26,27,28]. An unsupportive and uncivil work environment has frequently been identified as one of the reasons that a nurse will leave a position and, more importantly, the profession entirely [29,30]. Our findings are consistent with others’ results indicating greater than 20% of participants reported leaving a position due to aggression.

The prevalence of incivility in the healthcare setting and general sources of incivility, as well as the impacts of incivility on RNs, other healthcare providers, patients, and families, has been previously reported [29,31]. RNs represent the largest number of staff providing care to patients and families in hospital settings [32]. RNs typically work very closely with other RNs and UAP team members providing critical hands-on care to patients on a day-to-day basis. Their ability to work together in a civil manner has a profound impact on patient outcomes and overall provider well-being. Input from RNs and UAPs must be considered when developing strategies to address incivility.

Qualitative responses indicate that leadership and peers need to treat one another with respect, kindness, and dignity. It was suggested that simply greeting others with positive body language and verbal acknowledgment fosters a culture of civility. An article in *Nurse Leader* supports the assertion that the lack of a simple hello is a dissatisfier for staff [33]. Additionally, the responses indicated that those in leadership positions need to be present and engaged and must address issues directly when they occur. Active listening and clear communication were described as behaviors demonstrating respect from management. Visibility and modeling of civil behavior by leaders was recognized as a crucial element to improve the working environment for staff and patients. In addition, the responses indicated that leadership and administration must actively support a no-tolerance policy for incivility and bullying. These comments were consistent with previous articles suggesting the importance of manager role modeling and support of no-tolerance policies as strategies to address incivility [10,34]. Finally, the comments suggested that reporting mechanisms and management must ensure that those reporting incivility do not face retaliation, a common concern frequently reported elsewhere [26,35].

## 8. Limitations

Self-selection bias and a decreased response rate may limit internal validity. At the time of data collection, the clinical staff reported staffing shortages, as well as a final surge of COVID-19 patients, that may have limited interest in participating in the study. Those that responded may disproportionately represent RNs and UAPs that have experienced incivility or those that have a greater working knowledge about incivility. Those that did not participate may represent a portion of the study population that are unclear about what constitutes incivility or are fearful of ramifications for reporting. The participants were predominantly white, non-Hispanic, female RNs. UAPs represented approximately 20% of respondents. The somewhat homogenous sample limits generalizability. Finally, this study was conducted in a large metropolitan hospital system in the southeast. It is possible that the new RNs/UAPs only completed the quantitative portion of the survey, did not recognize incivility, and/or did not have examples of uncivil situations that were resolved in a positive manner. 

There were additional global, social, and organizational factors that may have impacted the results of this study. This survey was deployed during 2021, the height of the COVID-19 pandemic in the U.S. In addition, there was significant civil unrest across the U.S. as a whole and in the local study area particularly related to the events surrounding George Floyd and police brutality against racial minorities. Finally, during the data collection process the target organization for this study underwent a 75% turnover in nursing leadership. These factors may have impacted the overall participation rates, responses, and results of this study.

## 9. Conclusions

The data collected in this study suggested that efforts, policies, and supportive strategies aimed specifically at RNs and UAPs have the potential to impact the rates of uncivil behaviors among and between RNs and UAPs. The identified differences in RN and UAP reporting suggest that educational efforts should be tailored specifically to enhance UAP recognition of covert acts of aggression and factors that contribute to incivility in the workplace. The educational needs of RNs and UAPs may differ; thus, varied approaches aimed at meeting the needs of these individual groups are needed. Additionally, strategies that include leadership development and system-/hospital-wide approaches must be developed to assist and support nursing staff when dealing with incivility perpetrated by patients, families, and physicians.

Future research should continue to focus on the specific sources of incivility, as well as identification of the knowledge and skills necessary for organizations and leadership to effectively maintain a positive work environment. Impactful educational and prevention strategies need to be developed, implemented, and most importantly evaluated for effectiveness. Inclusion of staff RNs and UAPs in the development and evaluation of prevention programs would be valuable and is highly recommended. Simulation is an approach that has strong potential to provide RNs, UAPs, and hospital leadership with needed opportunities to practice and develop skills to address and manage incivility. Finally, organizational systems that create effective reporting mechanisms, as well as no-tolerance policies for uncivil behavior, should be a cornerstone of all programs instituted to address incivility in the healthcare setting. An argument could be made for the consideration of standard reporting mechanisms across healthcare work environments to inform further broader policy decisions to minimize and mitigate negative behaviors within healthcare work environments. With an increase in recognizing the value of positions such as chief well-being officers for many healthcare organizations a unique opportunity exists to develop, test, and implement innovative interventions to prevent and mitigate negative behaviors within healthcare environments, which would likely reduce the burnout experienced by healthcare workers.

Leaders, administrators, and educators are well poised to demonstrate positive, civil behaviors using role modeling and active engagement. An optimistic and supportive culture within clinical units and overall hospital environments is best created with intention and vigilance. Healthy work environments are molded, guided, and nurtured by those in leadership positions. Leaders need to be authentic, visible, and well prepared to provide appropriate support to RNs and UAPs in all areas of the healthcare industry. As the nursing profession faces growing challenges, it is critical that organizations set a hopeful, inclusive, and respectful tone in the healthcare setting. 

## Figures and Tables

**Table 1:** Demographic characteristics.

Characteristic	Mean (SD)
Age (n = 304)	39.47 (11.37)
Experience (in years) (n = 307)	13.31 (10.14)
	N (%)
Sex (n = 311)	
Female	278 (88.8%)
Male	33 (10.5%)
Role (n = 308)	
Registered nurse	231 (75%)
Patient care technician	57 (18.5%)
Licensed practical nurse (LPN)	14 (4.5%)
Therapy assistant	6 (1.9%)
Shift (n = 307)	
Day	219 (71.3%)
Evening	3 (1%)
Night	85 (27.7%)
Race (n = 304)	
White	259 (85.2%)
Black or African American	31 (10.2%)
American Indian or Alaska Native	4 (1.3%)
Asian Indian	1 (0.3%)
Filipino	8 (2.6%)
Vietnamese	1 (0.3%)
Ethnicity (296)	
Not Hispanic, Latino/a or Spanish origin	286 (96.6%)
Yes, Mexican American or Chicano/a	2 (0.7%)
Yes Puerto Rican	3 (1%)
Yes, another Hispanic, Latino/a or Spanish origin	5 (1.7%)
Certification (n = 222)	
Yes	106 (47.7%)
No	116 (52.3%)
Degree (n = 426)	
None	2 (0.47%)
High school diploma	79 (18.5%)
Associate degree	77 (18.1%)
Bachelor of science (BS)	50 (11.7%)
Batchelor of science in nursing (BSN)	153 (35.9%)
Master’s degree	25 (5.9%)
Master of science in nursing (MSN)	34 (8%)
Doctor of nursing practice (DNP)	3 (0.7%)
Doctor of nursing science (DNS)	1 (0.2%)
Doctor of philosophy (PhD)	2 (0.5%)

**Table 2:** Mann–Whitney U results by Role.

Scales	Group	n	Mean Rank	Sum of Ranks	U	*p*-Value
**Negative Behaviors in Healthcare Subscales**						
Contributing factors (α = 0.90)	Nurse UAP	233 61	153.6 124.3	35,783.5 7581.5	5690.5	0.02
Observes aggression (α = 0.76)	Nurse UAP	232 61	154.7 117.6	35,899.5 7171.5	5280.5	0.001
Uses aggression (α = 0.84)	Nurse UAP	232 59	148.9 134.6	34,547.0 7939.0	6169.0	0.24
Recipient of aggression (α = 0.80)	Nurse UAP	237 62	155.8 128.0	36,914.5 7935.5	5982.5	0.02
Fears retaliation (α = 0.95)	Nurse UAP	238 62	150.5 150.6	35,812.0 9338.0	7371.0	0.99
**Nursing Incivility Scale Behaviors Subscales**						
Hostile climate (α = 0.88)	Nurse UAP	207 50	133.4 110.7	27,619.0 5534.0	4259.0	0.05
Inappropriate jokes (α = 0.94)	Nurse UAP	215 50	133.0 133.2	28,584.5 6660.5	5364.5	0.98
Inconsiderate behavior (α = 0.75)	Nurse UAP	209 51	133.8 117.1	27,959.5 5970.5	4644.5	0.15
Gossip and rumors (α = 0.94)	Nurse UAP	208 50	135.6 104.2	28,201.5 5209.5	3934.5	0.01
Freeriding (α = 0.91)	Nurse UAP	210 46	129.3 124.8	27,153.0 5743.0	4662.0	0.71
Abusive supervision (α = 0.85)	Nurse UAP	212 48	136.1 105.5	28,852.5 5077.5	3901.5	0.01
Lack of respect (α = 0.87)	Nurse UAP	205 47	129.1 115.4	26,456.5 5421.5	4293.5	0.24
Displaced frustration (α = 0.92)	Nurse UAP	213 50	131.3 135.0	27,968.0 6748.0	5177.0	0.76
**Nursing Incivility Scale Sources Subscales**						
General incivility (α = 0.87)	Nurse UAP	207 50	131.2 119.8	27,163.0 5990.0	4715.0	0.33
Nurse incivility (α = 0.93)	Nurse UAP	214 49	134.7 120.2	28,824.0 5892.0	4667.0	0.23
UAP incivility (α = 0.94)	Nurse UAP	203 45	129.1 104.0	26,197.5 4678.5	3643.5	0.03
Direct supervisor incivility (α = 0.94)	Nurse UAP	212 50	130.4 136.3	27,636.5 6816.5	5058.5	0.60
Physician incivility (α = 0.93)	Nurse UAP	210 48	137.4 94.8	28,862.5 4548.5	3372.5	<0.001
Patient and family incivility (α = 0.94)	Nurse UAP	210 47	129.4 127.4	27,164.0 5989.0	4861.0	0.87

Cronbach’s alpha for each subscale calculated including both RNs and UAPs.

## Data Availability

The data presented in this study are available on request from the corresponding author. The data are not publicly available to protect participant privacy.

## Public Involvement Statement

No public involvement in any aspect of this research.

## Guidelines and Standards Statement

This manuscript was drafted against the Checklist for Reporting of Survey Studies (CROSS) [36].
